# Acknowledgement to Reviewers of *Polymers* in 2019

**DOI:** 10.3390/polym12010172

**Published:** 2020-01-09

**Authors:** Polymers Editorial Office

**Affiliations:** MDPI, St. Alban-Anlage 66, 4052 Basel, Switzerland

The editorial team greatly appreciates the reviewers who have dedicated their considerable time and expertise to the journal’s rigorous editorial process over the past 12 months, regardless of whether the papers are finally published or not. In 2019, a total of 2109 papers were published in the journal, with a median time to first decision of 14 days and a median time from submission to publication of 35 days. The editors would like to express their sincere gratitude to the following reviewers for their generous contribution in 2019:
Aarstad, OlavLiu, ShanqinAbashev, George G.Liu, Shi-XiaAbate, LorenzoLiu, ShuangAbbina, SrinivasLiu, ShumeiAbbrent, SabinaLiu, Shyh-JiunAbe, MitsuruLiu, SongpoAbel, Steven M.Liu, TaihongAbenojar, JuanaLiu, TianyuAbitbol, TiffanyLiu, Ting-YuAbuin, GracielaLiu, TongAbushammala, HatemLiu, WeidongAchilias, Dimitris S.Liu, WendiAdachi, NaoyaLiu, Xian HuAdamopoulos, StergiosLiu, XianhuAdamson, Douglas H.Liu, XiaoboAdamus, GrazynaLiu, XifengAdel, PenhasiLiu, XuguangAdhikari, Birendra B.Liu, XuqingAdonin, Sergey A.Liu, YaminAflori, MagdalenaLiu, YaodongAfzal, AdeelLiu, YijiangAgarski, BorisLiu, YijingAgarwal, AnimeshLiu, Ying-LingAgrawal, RichaLiu, YingtaoAguayo, Maria GracielaLiu, YuAguilar-Bolados, HectorLiu, Ze-huaAhmad, ZeeshanLiu, ZhiAhmed, MaryaLiu, ZhitianAhmed, SalehLiu, ZitongAida Alejandra, Perez-FonsecaLizardi-Mendoza, JaimeAimi, JunkoLizundia, E.Aizawa, TakafumiLizundia, ErlantzAk, MetinLogothetidis, StergiosAkiba, IsamuLondono Lopez, Martha ElenaAkira, HafukaLong, BrianAkitsu, TakashiroLonghi, GiovannaAkopova, TatianaLopez, FrancescoAla, GuidoLopez-Barron, Carlos R.Alba, MariaLopez-Cuesta, Jose-MarieAl-Bataineh, SameerLopez-Garzon, F.J.Albertin, LucaLopez-Lorente, Angela InmaculadaAlbo, JonathanLoppinet, BenoitAlcock, BenLorandi, FrancescaAlcoutlabi, MatazLorenz, AlexanderAleshin, AndreyLoretz, BrigittaAlexandrova, LarissaLosada-Perez, PatriciaAlhwaige, AlmahdiLosio, SimonaAli, Md AzaharLou, Ching-WenAli, SamimLou, ShuaiAli El-Remaily, MahmoudLou, YanAli Khan, MoonisLou, ZhichaoAli Raza, ZulfiqarLow, NicholasAli Shah, Anwar-ul-HaqLu, FachuangAlkan, CemilLu, JingzhiAllegra, MarioLu, XianyongAlmeida, HugoLu, YanAlmeida Junior, Jose HumbertoLubczak, JacekAlongi, JennyLuda, Maria PaolaAlosmanov, RasimLuhrs, Claudia C.Altan, MirigulLui, Wai-BunAltarawneh, MohammednoorLukasik, RafalAltstädt, VolkerLukomska-Szymanska, MonikaAlvarez, Alejandro J.Luliński, PiotrAlvarez, JoaquinLuna, CarlosAlves, LuisLuo, MengboAlves, NunoLuo, RongcongAmarowicz, RyszardLuo, ShaojuanAmbrogi, VeronicaLuo, XiaolinAmbrosio-Lazaro, Roberto CarlosLuo, YanhuaAmer, HassanLuo, YunjunAmii, HidekiLuong, Dũng (Dzung) DinhAmine, AzizLupu, StelianAmsden, BrianLuukkonen, TeroAmza, CatalinLuyt, AdriaanAn, HyosungLuz, Sandra MariaAn, JiaLuzi, FrancescaAn, QiLyons, JohnAn, SeongpilLyu, HailongAnanthakrishnan, Soundaram JeevarathinamLyulin, Alexey V.Andablo-Reyes, Efren AlbertoM Gomez, ClaraAndreopoulou, Aikaterini K.Ma, JianzhongAndritsch, ThomasMa, JieAndrzejewska, EwaMa, LiangAndrzejewski, JacekMa, MingguoAngellier-Coussy, HelèneMa, TianyiAngione, Maria DanielaMa, ZhongqingAnnamalai, Pratheep KumarMachado, FabricioAnselmet, FabienMadden, DavidAntal, DianaMaeda, TomokiAntico, EnriquetaMaeyama, TakuyaAntipina, MariaMagalhães, Fernão D.Antonioli, DiegoMagasinski, AlexandreAntunes, AnaMagkoev, Tamerlan T.Antunes, MarceloMagyari, KlaraAnyszka, RafalMahmood, HaroonAoki, DanMaietta, SaverioAota, HiroyukiMaior, IoanaAoyagi, TakeshiMajor, IanApetrei, ConstantinMaksimkin, AlekseyApostoluk, AleksandraMakvandi, PooyanAppleby-Thomas, GarethMalanga, MiloArakawa, YukiMaldonado, Jose-LuisAranguren, Mirta I.Male, UmashankarAraya-Hermosilla, RodrigoMalgorzata, MatusiakArdelean, Lavinia CosminaMalik, Muhammad ImranAres-Pernas, AnaMalinauskas, MangirdasArgüelles-Monal, Waldo M.Malinowski, SzymonAriga, KatsuhikoMallamace, DomenicoArinstein, ArkadiMallamace, FrancescoArjmand, MohammadMallavia, RicardoArjmandi, RezaMalmstadt, NoahArkas, MichaelMalucelli, GiulioArnaud, FrançoiseMamane, VictorArrieta, MarinaMamiński, MariuszArrigo, RossellaMan, AdrianArsene, CeciliaManakhov, AntonArshad, AdeelManca, Maria LetiziaArslan, HakanMandal, AjayAsai, DaisukeMandracchia, DeliaAshley, JonManfredi, LuigiAssaf, Khaleel IbrahimMangalara, Jayachandra HariAta, SeisukeMania, SzymonAtabaev, TimurManian, Avinash P.Atanase, LeonardManiruzzaman, MohammedAtrei, AndreaMannari, VijayAtta-Obeng, EmmanuelMantione, DanieleAuclair, NicolasMao, LeiAudette, Gerald FMao, YiminAuriemma, FiniziaMarat-Mendes, RosaAurus, Rafael A.Marchesini, FlAvio H.Avila-Orta, Carlos AlbertoMarega, CarlaAvolio, RobertoMargaillan, A.Avramidis, StavrosMargheritis, EleonoraAyres, NeilMarić, MilanAytac, AyseMarin, EliaAytemiz, DeryaMarin, LuminitaAyukawa, YasunoriMarin-Genescà, MarcAyyavoo, JayalakshmiMarkowski, KonradAzizi, SohrabMarques, Ana ClaraAzzaroni, OmarMarques, CarlosBaba, KoumeiMarrocchi, AssuntaBabeva, TsvetankaMartella, DanieleBadea, IldikoMartikka, OssiBadia, JoseMartincic, EmileBae, Hae-RahnMartinelli, EnzoBae, Hyung D.Martinez De Ilarduya, AntxonBae, JoonwonMartinez-Hernandez, Ana LauraBae, Ki HyunMartinez-Perez, Carlos A.Bae, Tae-HyunMartinez-Tong, Daniel E.Baggiani, ClaudioMartinka, JozefBagi, PeterMartino, SabataBai, BofengMartinotti, SimonaBai, ChengyingMartin-Ramos, PabloBai, HongweiMartins, P. M.Bai, JiamingMartins Amaro, Ana Paula BetencourtBai, ShiqiangMarto, Carlos MiguelBaig, ChunggiMaruschak, PavloBaji, AvinashMarzec, AnnaBakar, MohamedMasania, KunalBalalaeva, Irina V.Masashi, IshikawaBalandin, Alexander A.Masato, DavideBalasingam, Suresh KannanMasayoshi, OkuboBalcar, HynekMascia, LenoBaldino, LuciaMasek, AnnaBaldino, NoemiMaslowski, MarcinBaljon, Arlette R. C.Massoud, SalahBallard, DavidMastrangeli, MassimoBallard, NicholasMasubuchi, YuichiBandari, SureshMasullo, MariorosarioBanerjee, Shib ShankarMatczak-Jon, EwaBao, ChunhuiMatei, AlinaBao, YongmingMatei, AndreeaBarabanova, Anna I.Mathew, ThomasBarandiaran, IratiMatko, VojkoBaranowska, HannaMatsidik, RukiyaBarany, TamasMatsui, HirosukeBarba, Francisco J.Matsumoto, KozoBarbera, JoaquinMatsumoto, MitsuharuBarczewski, MateuszMatsumura, KazuakiBarile, ClaudiaMatsumura, ShuichiBarille, RegisMatsuura, HiroshiBarra, GiuseppinaMatteis, Valeria DeBarra, Guilherme M.O.Matykiewicz, DanutaBarreca, SalvatoreMautner, FranzBarretta, RaffaeleMaver, TinaBarry, CarolMavromatidis, LazarosBarszczewska-Rybarek, IzabelaMazzanti, ValentinaBartoli, MattiaMazzocchetti, LauraBartolomeo, Antonio DiMcCoy, JohnBasnett, PoojaMcHugh, KevinBastakoti, Bishnu PrasadMechrez, GuyBastidas-Oyanedel, Juan-RodrigoMedel, Francisco JavierBastola, EbinMederic, PascalBataille, IsabelleMedronho, Bruno Filipe FigueirasBatchelor, HannahMeen, Teen-­HangBattegazzore, DanieleMees, MaartenBauer, FrancoisMehrali, MehdiBaumann, MarcusMehrali, MohammadBaumgarten, MartinMehrotra, Gopal KrishnaBavasso, IreneMeijide, FranciscoBayer, IlkerMekasha, SophanitBayraktar, EminMekonnen, TizazuBazaka, KaterynaMelinte, VioletaBazzaz, MohammadMen, YongjunBeachley, VinceMendrek, BarbaraBeard, James D.Meneguzzo, FrancescoBec, Krzysztof B.Meng, FanbenBedini, EmilianoMeng, FanbinBednar, PetrMeng, HongBednarz, SzczepanMeng, XiangchaoBehnood, AliMenger, Fredric MBelBruno, JosephMequanint, KibretBella, FedericoMeram, ChalamaiahBellantone, VincenzoMessimer, SherriBellisario, DeniseMessina, FabrizioBelsley, Michael ScottMezzomo Collares, FabricioBeltran, Ana M.Mi, DashanBeltran-Huarac, Juan C.Mia, MozammelBeltsios, K.Micheli, LauraBenelli, TizianaMichels, JulienBenes, HynekMichieletto, DavideBengtson, GiselaMichl, ThomasBeniah, GoliathMichl, Thomas D.Benitez Jimenez, Jose JesusMiculescu, FlorinBento, RitaMicutz, MarinBeris, AntonyMielańczyk, AnnaBermeshev, MaximMiesbauer, OliverBernaerts, Katrien V.Miguel, Sonia P.Bernstorff, SigridMija, AliceBertani, RobertaMiki, TsunehisaBesagni, GiorgioMikulchyk, TatsianaBesford, QuinnMilanesio, MarcoBettotti, PaoloMilchev, AndreyBeuermann, SabineMilCius, DariusBhagia, SamarthyaMilionis, AthanasiosBhattacharjee, ArnabMiller, ReinhardBhattacharya, SupriyoMillett, JeremyBhaumik, AsimMiluski, PiotrBhowmik, Pradip K.Milyukov, Vasili A.Bialek, MarzenaMin, DaominBian, HuiyangMin, Se DongBian, LimingMinakshi, ManickamBieliński, Dariusz M.Minato, Ken-IchiroBiernasiuk, AnnaMintcheva, NeliBikiaris, DimitriosMiquelard-Garnier, GuillaumeBilal, SalmaMiranda, Jose M.Bilisik, K.Miranda, RubenBin, FeiMiravet, Juan F.Binder, KurtMirsayar, MirmiladBirleanu, CorinaMishra, Pawan KumarBisoyi, Hari KrishnaMishra, PriyankaBistricic, LahorijaMishra, YogendraBiswas, KaushikMistretta, Maria ChiaraBitsanis, IoannisMitropoulos, Alexander N.Bittolo Bon, SilviaMitsumata, TetsuBlanche, Pierre-AlexandreMitus, AntoniBlanco, AngelesMizera, AlesBlanco, IgnazioMizsei, JanosBleul, ReginaMoad, GraemeBlokhin, Alexander M.Moatsou, DafniBobbala, SharanMocanu, G.Bober, PatrycjaModukuri, RamkumarBobrovsky, AlexeyMohammed, Abdul SamadBoccia, Antonella CaterinaMohan, Velram BalajiBociąga, ElzbietaMohanta, AntaryamiBoda, Sunil KumarMoine, LaurenceBodaghi, MahdiMoineau-Chane-Ching, Kathleen I.Boffito, MonicaMolina, MariaBokias, GeorgiosMoliner, CristinaBoldt, RegineMolino, PaulBonadies, IreneMollica, FrancescoBonifAcio, VascoMomm, FelixBonilla-Cruz, JoseMoncho Jorda, ArturoBonnet, FannyMonfort, OlivierBonomo, MatteoMonkova, KatarinaBonyAr, AttilaMonroe, Mary Beth BrowningBoota, MuhammadMonteil-Rivera, FannyBorbas, K. EszterMonti, MarcoBorges, João PauloMoon, Doo KyungBormashenko, EdwardMoraczewski, KrzysztofBorriello, AnnaMorales-Vidal, MartaBorsdorf, HelkoMoratti, StephenBortner, MichaelMoratti, Stephen CBorzacchiello, AssuntaMoravCik, IgorBottari, FabioMoreirinha, CatarinaBou, Santiago FerrandizMorgan, AlexanderBouazizi, NabilMorganti, PierfrancescoBouckaert, JulieMori, WasukeBounor, VeroniqueMorikawa, AtsushiBourbigot, SergeMorimoto, NobuyukiBouwman, WimMorouço, PedroBowlin, GaryMorreale, MarcoBoy, RamizMoscicka-Grzesiak, HannaBoyd, AnthonyMotoyanagi, JinBoyd, DarrylMotta, AntonellaBracciale, Maria PaolaMoussaoui, YounesBrajlih, TomazMracek, AlešBrancart, JoostMrlik, MiroslavBranda, FrancescoMrowczyński, RadoslawBraslau, RebeccaMroz, ZenonBraunecker, WadeMrukiewicz, MateuszBrebu, MihaiMunguia, JavierBrecker, LotharMunoz-Bonilla, AlexandraBrennan, CharlesMunoz-Guerra, SebastianBriatico-Vangosa, FrancescoMunteanu, BogdanBrischetto, SalvatoreMurshid, NimerBroda, JanMusmarra, DinoBrooks, AmandaMust, IndrekBrosse, NicolasMusto, PellegrinoBrosseau, ChristianMuthuramalingam, ThangarajBrowar, Andrew W.Mutlu, HaticeBruijns, BrigitteMuziol, Tadeusz MikolajBrutti, SergioNa, LiBrychcy, EwaNaderi, G.Bryja, Leszek BryjaNadolny, ZbigniewBrzeska, JoannaNadykto, Alexey B.Brzezinski, MarekNaebe, MaryamBrzozka, AgnieszkaNaga, NaofumiBuaki-Sogo, MireiaNagai, HirokiBucio, EmilioNah, Jae-WoonBuckner, StevenNahar, LamiaBudnyak, TetyanaNair, Devatha P.Bui, Thanh-TuAnNair, Jijeesh RaviBui, Tinh QuocNair, RemyaBuijs, WimNair, Sandeep SudhakaranBuj Corral, IreneNair, VaishakhBujanovic, BiljanaNakagaito, Antonio NorioBulgariu, LauraNakano, HideyukiBumgardner, Joel D.Nakano, MasahiroBurkhardt, TheodoreNakas, James P.Buscaglia, MarcoNakata, NorioByun, JeehyeNakayama, YuushouCabanelas, Juan CarlosNakhmanson, Serge M.Cabot, Pere L.Namazi, HassanCabrales, LuisNameni, GordonCabulis, UgisNamiesnik, JacekCacciotti, IlariaNanaki, StavroulaCademartori, PedroNand, AshveenCadenas Pliego, GregorioNandi, ChayanCadinoiu, Anca NiculinaNarayanan, GaneshCaggiano, AntonioNardecchia, StefaniaCai, ChaoNarducci, RiccardoCai, GuofaNash, Michael A.Cai, LipingNasiri, MohammadrezaCalabrò, VincenzaNatesan, ShanmugasundaramCalaon, MatteoNaumann, StefanCaldera, FabrizioNaumov, AntonCalvignac, BriceNavalon, SergioCalvo, LourdesNavarro, Francisco JavierCamara, Jose SousaNavarro, RodrigoCamba, A. TundidorNavarro Dominguez, Francisco JavierCametti, CesareNawarathna, DharmakeerthiCaminero Torija, Miguel AngelNaylor, AndrewCammas-Marion, SandrineNazare, ShonaliCanal, Jose MariaNazarova, DimanaCanavate, JavierNechyporchuk, OleksandrCandan, ZekiNedelchev, LianCangialosi, DanieleNeel, Ensanya A. AbouCanle, MoisesNeffe, Axel T.Cantarella, MariaNegahdar, LeilaCao, JianyunNeisiany, Rasoul EsmaeelyCao, YuheNemes, Norbert M.Cao, ZhenNemeş, OvidiuCapacchione, CarmineNerin, CristinaCaputo, PaolinoNesbitt, WarwickCaracciolo, VincenzoNeto, VictorCarapeto, AnaNetz, PauloCarbone, AlessandraNeufurth, MeikCardinaels, RuthNeugebauer, DorotaCardon, LudwigNg, Wei LongCarlos Salay, LuizNghia, Truong PhuocCarosio, FedericoNgo, Tri DungCarraro, MauroNguyen, Duc ThanhCarrasco, SergioNguyen, Ngoc A.Carrillo, WilmanNguyen, T. HienCarsi, MartaNguyen, Tran-Minh GiaoCarter, AlanNguyen-Tri, PhuongCaruso, GabriellaNicholson, John W.Carvalho, Luisa M. H.Nicoletta, Fiore PasqualeCasado-Coterillo, ClaraNie, ChuanxiongCaseri, WalterNiemczyk, AgataCasettari, LucaNieminen, KaarloCaspary, ReinhardNikolaidis, Alexandros K.Cassano, AlfredoNikolakakis, IoannisCastanheiro, JoseNilsson, FritjofCastell, PereNinomiya, SatoshiCastneiras, AlfonsoNischang, IvoCastro, Jose MNishikiori, HiromasaCastro-Munoz, RobertoNishikitani, YoshinoriCastro-Vargas, Henry I.Nishiyabu, RyuheiCataldi, PietroNitulescu, MihaiCatapano, AnitaNobile, LucioCavalcanti Da Silva Filho, EdsonNoh, SeungmanCavallaro, GiuseppeNomura, KotohiroCavallieri, Angelo Luiz FazaniNonoyama, TakayukiCavallotti, CarloNoor, NuruzzamanCavicchi, KevinNoordermeer, Jacques W. M.Caydamli, YavuzNorrman, JensCecilia, LealNorvez, SophieCelik, EmrahNosker, ThomasCerbu, CameliaNouranian, SasanCerna, JorgeNoureddine, AchrafCernik, MiroslavNouvel, CecileCerrada, Maria LuisaNovio, FernandoCerveny, SilvinaNowaczyk, JacekCervino, GabrieleNowak, WojciechCesano, FedericoNumez, EstrellaCha, ChaenyungNuno V., GamaCha, Sang-HoNypelo, TiinaCha, Sung WoonOancea, FlorinChacko, Jenu VargheseObeng, Yaw S.Chacon Munoz, Jesus MiguelObermeyer, AllieChae, MichaelObruca, StanislavChalioris, ConstantinOchoa, Nelio ArielChan, Hon FaiOda, YukariChan, Michael C. W.Odelius, KarinChan, Yi-TsuOgam, ErickChandra, RameshOgnyanov, ManolChang, Dong WookOh, Dongyeop X.Chang, Jin-HaeOh, JonghyunChang, JiyoungOh, Min-cheolChang, MincheolOhara, KeishiChangzeng, YanOhsedo, YutakaChao, HuikuanOhta, YoshihiroCharas, AnaOikonomou, Evdokia K.Charles, LaurenceOjeda-Benitez, SaraChase, George G.Ojo, EbenezerChatterjee, SudiptaOkahisa, YokoChaubet, FredericOkamoto, MasamiChaudhari, NitinOkamoto, YoshiyukiChauhan, Ghanshyam SinghOkamura, HaruyukiChavali, MurthyOkay, OguzChavez, FermanOkoshi, MasayukiChavez-Angel, EmigdioOlek, WieslawCheaburu-Yilmaz, Catalina NataliaOlewnik-Kruszkowska, EwaChecchetto, RiccardoOliva, LeoneChehimi, MohamedOliveira, MonicaChemtob, AbrahamOliveira, SaraChen, ChengOliveira Lobo, AndersonChen, ChiachungOlmos, DaniaChen, ChuanpinOlofinjana, AyodeleChen, Chuh-YungOno, KyosukeChen, ChunhuiOnuta, Marie-ChristineChen, FengOnyshchenko, YuliiaChen, GuoOrte, AngelChen, Hsin-LungOrtenzi, Marco A.Chen, I-Wen PeterOrtiz, InmaculadaChen, Jem-KunOsakada, KohtaroChen, JiahaoOsmialowski, BorysChen, Jian-LianOsswald, TimChen, JinhuaÖsterholm, AnnaChen, JunOstrauskaite, JolitaChen, LiangOtero, MartaChen, LingxinOtero, T.F.Chen, Lung-ChienOuyang, Xiao-kunChen, MingshengOyedele, AkinolaChen, Po-chiaOzaki, YukihiroChen, Roland K.Ozaltin, KadirChen, XiangzhongPacheco-Catalan, DaniellaChen, XinPaciorek-Sadowska, JoannaChen, Yi-ChunPadamati, Ramesh BabuChen, Yi-WenPadil, Vinod V. T.Chen, Yong-SongPadilla, JavierChen, YuanfuPagacz, JoannaChen, Yu-ChunPakdel, EsfandiarChen, Zhi-GangPalamà, IlariaChen, ZhongPalardy, GenevieveChen, ZhongbingPalermo, EdmundChenal, Jean-MarcPalinko, IstvanCheng, Chih-ChiaPalkova, HelenaCheng, GangPaluch, Andrew S.Cheng, H. N.Pan, CaiyuanCheng, JiangPan, GuoqinCheng, JuePan, GuoqingCheng, LishengPan, HaihuaCheng, PeiPan, NingCheng, ZhiqiangPan, QinCheong, In WooPan, YuanfengCheung, OceanPanaitescu, Denis M.Chiang, Chin LungPandey, SadanandChiang, Long YPanek, MilošChiang, Yi-TingPankow, MarkChiang, Yu-ChunPannerselvam, SureshChiappone, AnnalisaPantani, RobertoChiessi, EsterPantano, MariaChiu, Chih-WeiPantoya, MichelleChiu, Fang-ChyouPanzarasa, GuidoChizhik, Alexey I.Paolino, MarcoChladek, GrzegorzPapa, IlariaChlopek, JanPapadakis, RaffaelloChmielarz, PawelPapadimitriou, VassilikiCho, ChungyeonPapadopoulos, Antonios N.Cho, HyunahPapageorgiou, George Z.Cho, SunghunPapagiannopoulos, AristeidisChoi, Hyo-JickPapastergiou, MariaChoi, Jae-HongParajuli, DurgaChoi, PhillipParamo-Garcia, UlisesChong, LiParaskevopoulou, PatrinaChou, Shih-FengParedes-Madrid, LeonelChowdhury, MohammadParis, JuanChremos, AlexandrosParisi, FilippoChristova, DarinkaParisini, EmilioChronopoulou, LauraPark, Deog-SuChrusciel, Jerzy J.Park, JeyoungChrzan, Krystian LeonardPark, Ji-WonChu, Yen-hoPark, Jong-SeokChua, BeeleePark, JuhyunChua, Chee KaiPark, Seon JooChuang, Er-YuanPark, Soo-JinChung, Chan I.Park, Su-IlChung, Chan-MoonPasbakhsh, PooriaChung, Hyun-JoongPaseiro Losada, PerfectoChung, Neal Tai-ShungPasparakis, GeorgiosChung, Sheng-HengPassaglia, ElisaChung, Shin HyePastor, Jose L.Chung, Shyan-LungPastore, RobertoCiapetti, GabrielaPaszkiewicz, SandraCiardelli, FrancescoPatel, JigneshkumarCicala, GianlucaPatra, NiranjanCiesielski, MichaelPatrickios, C.Cifre, Jose G. H.Pattamaprom, CattaleeyaCinteza, Otilia LudmilaPatterson, JenniferCiolacu, DianaPauer, WernerCipullo, RobertaPaul, CharlesCirillo, GiuseppePauliac-Vaujour, EmmanuelleCitterio, BarbaraPauliukaite, RasaCivera, Maria ConcepcionPaulsen, HaukeClare, Tami LasseterPaun, Maria-AlexandraClochard, Marie ClaudePavic, ValentinaCochis, AndreaPavliC, MatjazCody, DervilPeer, AkshitCoelho, PauloPeer, PetraCoelhoso, IsabelPeeters, RoosCoghe, FrederikPegoretti, AlessandroCohen Stuart, MartienPei, EujinCoiai, SerenaPei, ZhiqiangCojocaru, CorneliuPellis, AlessandroColeman, AnthonyPeng, Chi-HowCollar, Emilia P.Peng, Robert Y.Collins, MauricePeng, XiaohongColom, XavierPeng, XinshengCompan Moreno, VicentePengcheng, LiComuzzi, ClaraPenkova, Anastasia V.Concilio, SimonaPerale, GiuseppeCong, TaoPerego, ClaudioConstantinescu, Dan MihaiPereira, Anselmo SoeiroConte, ClaudiaPereira, RubenConti, BicePerez, EliasConzuelo, FelipePerez Munoz, AntonioCook-Chennault, KimberlyPerez-Alvarez, L.Cools, PieterPerez-Köhler, BarbaraCooney, RalphPerez-Mendoza, Manuel J.Coppola, BartolomeoPerinelli, Diego RomanoCorcione, Carola EspositoPerumal, SugunaCordeiro, Rosemeyre A.Pester, ChristianCorigliano, AlbertoPestov, AlexanderCorradi, MarcoPetcu, CristianCorrao, RossellaPeterson, BrandonCorray, ColinaPeterson, Gregory I.Correia, IlidioPetrov, P.Corrigan, Damion KPetrović, MarijaCorrigan, NathanielPetrucci, RobertoCortaberria, GalderPettarin, ValeriaCortes, Farid B.Pezzin, SergioCortez-Lemus, Norma AidePham, JonathanCorvo, Marta C.P.C.Pham, Tien DucCosma, PinalysaPham, TungCosta, FrancoPhan, Hoang-PhuongCosta, Jose Arnaldo SantanaPhoenix, S. LeighCosta, V.A.F.Piedade, Ana PaulaCoutinho, Paulo Jose GomesPielichowska, KingaCovas, Jose AntonioPielichowski, KrzysztofCovino, RobertoPiemontesi, FabrizioCraciun, GabrielaPierini, FilippoCrane, NathanPietrasik, JoannaCretich, MarinaPiletsky, SergeyCristescu, RodicaPilyugina, EkaterinaCristian-Dragos, VarganiciPinalli, RobertaCroitoru, CatalinPinho, LuisCroll, Andrew B.Pinson, JeanCrucho, Carina Isabel CorreiaPinteala, MarianaCruz, Maria-LuisaPinto, JavierCruz, SandraPinto, RicardoCsihony, SzilardPinto, SandraCudzilo, StanislawPinto Da Silva, LuisCui, HaitaoPintor, ArianaCui, WenguoPiras, Anna MariaCulebras, MarioPirzada, TahiraCybulska, JustynaPiskin, SenolCzarnecka-Komorowska, DorotaPispas, StergiosD’Annibale, FrancescoPistone, AlessandroDa Conceicao, Thiago F.Piszczyk, lukaszDa Motta, M.Pitsikalis, MarinosDa Silva, Emerson RodrigoPitto-Barry, AnaïsDa Silva, MarceloPiunova, VictoriaDalle Vacche, SaraPizzi, AntonioDama, MuraliPlacha, DanielaDamkaci, FehmiPlasseraud, LaurentD'Amora, UgoPlonska-Brzezinska, MartaDan, DarrenPlotka-Wasylka, JustynaDan, DobrotaPoças, FatimaDa'na, EnshirahPochard, IsabelleDanko, MartinPoddel'sky, AndreyDante, SilviaPodgornik, RudiDanti, SerenaPoetschke, PetraDargazany, RoozbehPoinsot, VerenaDarvell, Brian W.Polanski, MarekDas, MahuyaPoletto, MatheusDasari, AravindPolimeni, AntonellaDash, BirajaPolitakos, NikolaosDaus, AlwinPolitano, AntonioDave, SandeepPoma, AlessandroDavi, FabrizioPonnamma, DeepalekshmiDavis, Eric M.Pons, JosefinaDawes, JudithPontikoglou, CharalamposDawn, ArnabPopa, LacramioaraDe Caro, VivianaPopielarz, RomanDe Deene, YvesPorcarelli, LucaDe Falco, GianluigiPorzionato, AndreaDe Grooth, JorisPosadas, PilarDe La Flor, SilviaPospiech, DorisDe La Torre, Jose GarciaPosselt, DortheDe Laporte, LauraPotapov, AndreiDe Leon, A. S.Pouponneau, PierreDe Luca, AlessandroPourrahimi, Amir MasoudDe Marco, CarmelaPrabhu, VivekDe Marco, IolandaPradanos, PedroDe Moraes, Ana CMPradhan, ShantanuDe Oliveira Barra, Guilherme MarizPragliola, StefaniaDe Paz, Antonio TaberneroPraprotnik, MatejDe Santis, RobertoPrashar, SanjivDe Simone, Salvatore GiovanniPraticò, Filippo GianmariaDe Sousa, Frederico B.Predoi, DanielaDe With, GijsbertusPresa Soto, M. AlejandroDe Zordi, NicolaPretsch, ThorstenDeceglie, MichaelPrhashanna, AmmuDehghanghadikolaei, AmirPrice, GarethDekel, Dario R.Pricl, SabrinaDel Barrio, JesusProfire, LenutaDel Hoyo Martinez, CarmenProkic, VesnaDel Rio, GabrielProsa, MarioDelattre, CedricProto, AndreaDelbecq, FredericPruitt, LisaDelfs, Jens-OlafPruna, Alina IulianaDelmotte, LucPrzybylek, PiotrDelogu, GiovannaPszczola, MarekDelpouve, NicolasPu, KanyiDemesa, Abayneh GetachewPucci, AndreaDemey, HaryPuchades, IvanDemin, AlexanderPuchalski, MichalDeng, RenhuaPuglia, DeboraDeng, ShengweiPuig, RitaDeng, XiaoranPuiggali, JordiDequidt, AlainPullano, SalvatoreDergunov, SergeyPulpytel, JeromeDerkatch, SvetlanaPunta, CarloDeRosa, ChristopherPunzo, FrancescoD'Errico, GerardinoPuszka, AndrzejDeshmukh, RajendraQi, Yu-HongDewapriya, NuwanQian, Jin-yuanDhakal, SomaQian, ShaopingDhakshinamoorthy, AmarajothiQiang, ZheDhaliwal, GurjotQin, CaiqinDhamodharan, RaghavachariQin, SanboDhand, VivekQiu, QiwenDhilip Kumar, Sathish SundarQu, Jin-PingDhindsa, Kulwinder SinghQu, MuchaoD'hooge, Dagmar R.Queiroz, Maria EugeniaDI, ChukovQuemener, DamienDi Girolamo, RoccoQuintana, RobertDi Maio, LucianoQuirino, Rafael L.Di Noia, Luigi PioR. Wellen, Renate M.Di Noto, VitoRabnawaz, MuhammadDiaz-Rodriguez, PatriciaRace, MarcoDiban, NazelyRadoev, BoryanDickert, Franz L.Rae, P.J.Diddens, DiddoRafiee, MohammadDiez-Pascual, Ana MariaRaftopoulos, Konstantinos NDimakopoulos, YannisRagauskas, ArthurDinca, ValentinaRahman, Md AnisurDing, BinRahman, Mohammad MizanurDing, ChunbangRahmani, OmeidDing, JianxunRahme, KamilDing, LiyunRaimondo, MarialuigiaDing, Wei DanRajauria, GauravDing, YifuRajendran, Jagathesh ChandraDingemans, TheoRamakrishnan, SathishDinis, Maria De LurdesRamakumar, KinthadaDinu, Maria ValentinaRamamoorthy, Sunil Kumar LindströmDiomede, FrancescaRaman, NamrataDionysopoulos, DimitriosRamanavicius, ArunasDobrotă, DanRamani, AlwarDobrzynski, PiotrRamezanzadeh, BahramDobrzyński, MaciejRamiasa, MelanieDockhorn, RonRamirez, JorgeDocoslis, ArisRamos, Ana P.Doddamani, MrityunjayRamos, JavierDodiuk, HannaRamos-Ortiz, GabrielDoeven, EganRanc, VaclavDöhler, DianaRangou, SofiaDoi, MasaoRanjkesh, AmidDomb, AviRanucci, ElisabettaDomenici, ValentinaRanzato, EliaDominguez Robles, JuanRao, AshitDominici, FrancoRao, QiDon, Trong-MingRapa, MariaDoroudiani, SaeedRaposo, MariaDos Santos, Demetrio JacksonRapson, Trevor D.Dou, JinboRascon, AgustinDoustkhah Heragh, EsmaeilRastogi, Vibhore KumarDrache, MarcoRaudino, AntonioDragostin, Oana MariaRault, FrançoisDrain, Charles MichaelRauwel, ProtimaDriscoll, MarkRavelli, DavideDrummond, CarlosRay, SudipDu, BoxueReany, OferDu, ChunfangRebollar, EstherDu, GuanbenRedondo, BelenDu, HaifengRedshaw, CarlDu, HaiyanRee, MoonhorDu, XushengRege, AmeyaDuan, BinReglero Ruiz, Jose AntonioDubey, Deepak KumarReifsnider, Kenneth L.Dudek, GabrielaReniers, FrançoisDuhamel, JeanRennhofer, HaraldDumee, LudovicRety, StephaneDunderdale, Gary J.Reyes-Ortega, FelisaDunky, ManfredRezaei-Gomari, SinaDurazzo, AlessandraŘezanka, MichalDutkiewicz, MichalRhee, Jin-KyuDzolic, ZoranRibeiro, Adriana SantosEbara, MitsuhiroRicci, GiovanniEbbesen, MortenRichtera, LukašEcheverria, CoroRick, Steven W.Ecker, MelanieRider, Andrew N.Edmondo, BattistaRiedel, Sebastian L.Edwards, BrianRiera-Galindo, SergiEgan, Paul F.Riess, GisbertEggenhöffner, RobertoRigamonti, LucaEhrmann, AndreaRigano, LuigiEjfler, JolantaRighetti, Maria CristinaEkielski, AdamRijavec, TatjanaElamin, KhalidRimeika, RomualdasElbert, JohannesRitchie, ChrisElder, SteveRitter, HelmutElena, Tiuc AncuţaRiveiro, AntonioElfalleh, WalidRivera-Armenta, Jose LuisEl-hadi, Ahmed M.Rizza, GiancarloElhag, SamiRobertson, ChristopheElias, AnastasiaRobles, EduardoElias BrAs, Ana RitaRocha, Hugo Alexandre OliveiraElias-Zuniga, AlexRochani, AnkitEliseeva, TatianaRocio-Bautista, PriscillaElizalde, Luis E.Rodrigue, DenisElmas, SaitRodrigues, AlirioEl-Safty, SherifRodriguez, Hortensia M.ElSherbiny, IbrahimRodriguez, MarioEndo, HitoshiRodriguez, NoelEndruweit, AndreasRodriguez Bernaldo De Quiros, AnaErcole, FrancescaRodriguez-Lorenzo, LuisEric, TillmanRogers, SimonErukhimovich, IgorRogers, Simon A.Erwan, PaineauRogulska, MagdalenaEscorihuela, JorgeRoh, ChanghyunEsen, CemalRohani, RosiahEslami, HosseinRojo, LuisEslamian, MortezaRoland, C. MichaelEsmaeely Neisiany, RasoulRoman R., StepanyanEspinosa, EduardoRomani Perez, AloiaEspinosa-Andrews, HugoRomanszki, LorandEsposito Corcione, CarolaRonca, AlfredoEssawy, HishamRonca, SaraEstelle, PatriceRondeau-Gagne, SimonEsteves, Bruno MRonen, AvnerEstrany, FrancescRoppolo, IgnazioEtienne, MathieuRosa, MarcoEtxeberria, AgustinRosenkranz, AndreasEvans, ChristopherRossella, DoratiEvtugyn, GennadyRossi, FilippoFajardo, Andre R.Rosu, DanFally, MartinRotaru, AndreiFan, Huan-JungRoth, Bernhard WilhelmFan, JizhouRoth, PeterFan, QinguoRottler, JoergFan, WeiRousakis, TheodorosFan, WeizhengRoy, SagarFang, ChengRoy, SwarupFang, Hsu-WeiRozwadowski, TomaszFang, Jia-YouRozynek, ZbigniewFang, LiangRubino, Federico MariaFapyane, DebyRubio, Leire RuizFare, SilviaRubio, Ramon G.Fariborz, KargarRubio, Ramon GonzalezFarris, StefanoRudawska, AnnaFarrokhpay, SaeedRuff, AdrianFarrugia, BrookeRuiz De Ballesteros, OddaFasano, VitoRusso, LauraFatehi, PedramRusso, PietroFatyeyeva, KaterynaRusso, TeresaFeczko, TivadarRybiński, PrzemyslawFei, BinRydz, JoannaFelgueiras, Helena P.Rytlewski, PiotrFeng, ChuangRytöluoto, IlkkaFeng, HongboRyu, SangjinFenyvesi, EvaRyu, SeongwooFerhan, Abdul RahimRyu Cho, Yu KyoungFerji, KhalidSabatini, ValentinaFernandes, Emanuel M.Saçak, MehmetFernandes, FabioSacchi, MarcoFernandes, IsabelSada, KazukiFernandes, Mariana Sofia PeixotoSadati, MonirosadatFernandez, Maria DoloresSadeghifar, HasanFernandez-Colino, AliciaSadovskaya, IrinaFernandez-Mayoralas, AlfonsoSaeb, Mohammad RezaFernandez-Romero, Juan ManuelSaha, BiswajitFerrante, ClaudioSaharan, LalitaFerreira, Ana MarinaSahoo, SangramaFerreira, FilipeSai, HiroakiFerreira, MarceloSain, SunandaFerreon, JosephineSaito, TakuyaFerrer, BelenSajdak, MarcinFerrero, Guillermo A.Sajid, MuhammadFerruti, PaoloSajkiewicz, PawelFerry, LaurentSakaebe, HikariFidalgo, MariaSakai, TadamotoFiedler, BodoSakai, TakenobuFigueira, RitaSakai, ToshioFigueira, Rita BacelarSakthivel, TamilselvanFiguereido Neto, AntonioSakurai, KazuoFilarowski, AleksanderSaladino, RaffaeleFilip, PetrSalado, ManuelFilipecki, JacekSalaoru, IuliaFilippone, GiovanniSalas-de La Cruz, DavidFiliz, VolkanSalasińska, KamilaFina, AlbertoSalaün, FabienFini, PaolaSalehiyan, RezaFiorelli, JulianoSalerno, SimonaFiori, StefanoSalis, AndreaFiorio, RudineiSallam, Hossam El-DinFirlak, MelikeSalmeia, Khalifah A.Fischer, HolgerSalonen, LauraFlandin, LionelSalzano De Luna, MartinaFlege, StefanSamec, JosephFliedel, ChristopheSameoto, DanFlorczyk, StephenSamhaber, W. M.Focaroli, StefanoSamper, Maria DoloresFodor, CsabaSamperi, FilippoFormela, KrzysztofSamulionis, VytautasFornaini, CarloSanchez-Soto, MiguelFörsth, MichaelSAnchez-Vergara, Maria ElenaFortunato, GiuseppinoSandoughsaz, AminFoster, Christopher W.Sandri, GiuseppinaFraga-Lopez, FranciscoSanjust, EnricoFragoso, AlexSantamaria-Echart, ArantzazuFranco, LourdesSantana, Marianella HernandezFrancolini, IolandaSantarelli, Maria LauraFrank, LuizaSantoro, SergioFranke, RochusSantos, Jose MariaFranz, GerhardSantulli, CarloFreire, Juan J.Sanyal, OishiFrielinghaus, HenrichSanz, MikelFrigione, MariaenricaSanz-Garcia, AndresFrihart, CharlesSapi, AndrasFroeyen, MatheusSarasini, FabrizioFrontistis, ZachariasSarkar, Amit K.Fu, JunSarkar, Amit KumarFu, LiansheSarto, FrancescaFu, QiangSasai, YasushiFu, Shih-FengSashiwa, HitoshiFu, YaoSasmal, ChandiFuchise, KeitaSato, HideyukiFuensanta, MonicaSato, KimiyasuFukuda, TakeshiSavagatrup, SucholFuoco, AlessioSavy, DavideFuoco, TizianaSawada, ToshikiFurlani, MaurizioSawai, JunGaan, SabyasachiSbirrazzuoli, NicolasGabr, Mohamed H.Scala, AngelaGahleitner, MarkusScampicchio, MatteoGaidukovs, SergejsScaramuzza, MatteoGaitzsch, JensScarfato, PaolaGalatus, RamonaSchab Balcerzak, EwaGalbis, ElsaSchacher, FelixGalembeck, FernandoSchachner, JörgGalgani, MarioSchadt, MartinGalkin, M. (Maxim)Schaefer, JenniferGalkin, MaximScharnagl, NicoGallego, SergiSchartel, BernhardGallei, MarkusSchirhagl, RomanaGalukhin, AndreySchmid, JochenGamez-Perez, JoseSchmid, ManfredGanazzoli, FabioSchmid, MarkusGandolfi, Maria GiovannaSchmidt, BernhardGanewatta, MitraSchmidt, MichaelGao, BaoxiangSchnabel, ManuelGao, HuileSchnabel, ThomasGao, PeiyuanSchoedel, AlexanderGao, ShuangSchubert, StephanieGao, YachenSchulz, BurkhardGao, YangSchulze, MargitGao, YongfengSchwaminger, SebastianGao, YongshengSchweizer, ThomasGao, Yu (Tom)Scribante, AndreaGao, YunxiangSedlacek, JanGao, ZheSedlacik, MichalGarbovskiy, YuriySeelig, ThomasGarcia, AraceliSefat, FarshidGarcia, NuriaSegal-Peretz, TamarGarcia Cucalon, LorenaSeh, Zhi WeiGarcia-Alcaraz, Jorge LuisSeib, PhilippGarcia-Castello, Esperanza MariaSeide, GunnarGarcia-Deibe, AnaSekiguchi, YuGarcia-Gonzales, CarlosSekkar, VGarcia-Granada, Andres-AmadorSelene, Sepulveda-GuzmanGarcia-Gutierrez, Mari CruzSemenyuk, PavelGarcia-Marquez, AlfonsoSeo, Young-SooGarcia-Martinez, Jesus-MariaSeong, Dong GiGarcia-Martinez, Joaquin C.Serenko, OlgaGarcia-Ramos, Juan CarlosSerpooshan, VahidGarcia-Romero, A.Serra, AngelsGarcia-Salaberri, Pablo AngelSerrà, AlbertGarcia-Torres, JoseSerrano, Dolores R.Garg, AkhilSevercan, FerideGarofalo, EmiliaSeydou, MahamadouGarrill, AshleySeyedin, ShayanGarvey, ChristopherSeyyed Monfared Zanjani, JamalGashti, Mazeyar ParvinzadehSgouros, AristotelisGautam, RekhaShaayegan, VahidGautam, SiddharthShafiei, AliGazi, MustafaShafiei, MahnazGazo, RadoShah, Kwok WeiGe, Jun CongShaik, Mohammed RafiGe, QiShamonin, MikhailGe, TingShamsaei, EzzatollahGee, WilliamShan, XinGei, MassimilianoShanjani, YaserGentile, GennaroShankar, EswarGeorge, GejoShao, LuGeorge, GraemeShao, TaoGeorge, Thomas F.Shapter, JoeGeorgopanos, ProkopiosSharma, Piyush SindhuGerbaldi, ClaudioSharma, PriyankaGericke, MartinSharma, Sunil K.Ghahremaninezhad, AliSharopov, FarukhGhannad-Rezaie, MostafaShavandi, AminGheju, MariusShen, EricGherardi, MatteoShen, XiGhoneim, MohamedShen, XiaojunGhori, Muhammad UsmanShen, Yu-FangGhosh, DebanjanaShen, ZhiqiangGhoshal, SushantaSheri, MadhuGierszewska, MagdalenaShestakov, MikhailGigliotti, MarcoShi, HengchongGillanders, Ross NShi, JinsongGindl-Altmutter, WolfgangShi, KaiyuanGiorcelli, MauroShi, TongfeiGiorgini, LorisShi, WenGiorleo, LucaShi, YongqianGiovannoli, CristinaShi, ZengqianGiraud, Marie NoëlleShibasaki, YujiGiraud, StephaneShiddiky, MuhammadGirelli, Chiara RobertaShimasaki, ToshiakiGiri, GauravShimojo, MasayukiGirolami, MarcoShimomura, TakeshiGitsov, IvanShin, Crystal S.Gizdavic-Nikolaidis, MarijaShin, Eun JooGkourmpis, ThomasShin, Hye KyoungGlasser, Wolfgang G.Shin, Jae SupGlomm, Wilhelm RShinde, SudhirkumarGloria, AntonioShirvanimoghaddam, KamyarGlukhova, Olga E.Shishkovsky, IgorGo, Jeung SangShou, WanGockenbach, ErnstShrestha, ShikhaGoding, JosefShyr, Tien-WeiGoh, KunliSikorski, WojciechGoh, Suat HongSilantyeva, Elena A.Gohy, Jean-FrancoisSilva, Carla ManuelaGolaszewski, JacekSilva, Nuno H. C. S.Golewski, Grzegorz LudwikSilvestre, Armando J. D.Gomes, Ana P.Silvestri, MarcoGomes, Clara S. B.Simakova, AntoninaGomes, Luciana CalheirosSimari, CataldoGomez-Soberon, Jose ManuelSimha Martynkova, GrazynaGon, MasayukiSims, Ian M.Gonçalves, Lidia M. D.Sing, Swee LeongGonçalves, RenatoSingh, GautamGong, XiongSingh, RajeshGonzalez, SergioSingh, VivekGonzalez-Benito, JavierSingha, SubhankarGonzalez-Vera, Juan A.Sinha, AbhijeetGooneie, AliSinha, JasmineGopalakrishnan, GopanSinha Ray, SaikatGopalan, SaianandSinha Ray, SumitGordiichuk, PavloSinha-Ray, SumanGorkunov, Maxim V.Siracusa, ValentinaGoseki, RaitaSirvio, JuhoGourinat, YvesSitarz, MaciejGoyanes, AlvaroSixta, HerbertGrande, AntonioSkinner, JackGrande Tovar, Carlos DavidSkirtach, AndreGranet, RobertSkorik, Yury A.Grau, GerdSkwarek, EwaGravalidis, ChristoforosSlepicka, PetrGreco, AntonioSlomkiewicz, Piotr MarekGreen, Nicola H.Slomkowski, StanislawGrest, Gary S.Sloop, JosephGriffini, GianmarcoSmiatek, JensGriffiths, RianSmith, DawnGrigsby, Christopher L.Smith, MargaretGrijalvo, SantiagoSmith Callahan, Laura A.Grilc, MihaSmolka, PetrGritsan, Nina P.Smrek, JanGroch, PawelSmulders, MaartenGrochowicz, MartaSo, HongyunGröschel, AndreSo, Kwok-FaiGrossi, MarcoSo, Monica C.Groza, AndreeaSoares, Bluma GGu, ChunjuSobiesiak, MagdalenaGu, JunweiSobus, JanGu, XiaodanSoccio, MichelinaGu, YuweiSocol, MarcelaGuan, QingbaoSödergård, AndersGuazzelli, ElisaSohrabpoor, HamedGuedes, Rui MirandaSokolova, Maria P.Guenet, Jean-MichelSon, Seung UkGuessasma, SofianeSong, ChangsikGuigo, NathanaelSong, Guang-LingGuijun, XianSong, Jeong-HoonGuittard, FredericSong, JinshengGulicovski, JelenaSong, JuhaGumfekar, SarangSong, KenanGunkel, IljaSong, Sung HoGuo, BaolinSong, XuanGuo, BinSong, ZhiyiGuo, FangweiSong, ZiyuanGuo, JiangSonnier, RodolpheGuo, JianglongSorrentino, AndreaGuo, QiuquanSorrentino, LuigiGuo, Si-YaoSotta, PaulGuo, WeihongSousa, AnaGuo, Zhan-HuSousa, Sergio F.Guo, ZhanyongSperanza, VitoGuo, Zhao-XiaSpina, RobertoGuofei, ChenSpinelli, GiovanniGupta, AnjuSpirk, StefanGupta, MohitSpoerk, MartinGupta, SanjuSrebnik, SimchaGutekunst, Will R.Sreerama, SubramanyaGutierrez Ramirez, ManuelSrinivasan, GowrishankarGutt, GheorgheSriram, GopuGuzman, EduardoStachewicz, UrszulaGuzman-Puyol, SusanaStaicu, TeodoraHa, Min-WooStary, ZdenekHadjadj, AomarStasiak-Rozańska, LidiaHager, Martin D.Stathopoulos, CostasHaghshenas, Hamzeh F.Staubitz, AnneHahn, MariahStawski, DawidHai, TranSteele, Terry W.J.Hakli, OzgulStefan, MariusHamciuc, CorneliuStejskal, JaroslavHampp, NorbertStekovic, DejanHan, DezhiStelescu, Maria DanielaHan, EricStephanou, PavlosHan, GuangpingSterzyński, TomaszHan, XiuyouSteup, MartinHan, YousooStewart, BeverlyHanafy, Nemany A.N.Stewart, ToddHandge, Ulrich A.Stiriba, Salah-EddineHanif, AsadStöckelhuber, Klaus WernerHansen, UweStokke, Bjørn TorgerHansma, HelenStolarczyk, AgnieszkaHaponiuk, JozefStoleru, ElenaHara, MitsuoStolojan, VladHarasym, JoannaStorti, GiuseppeHardy, JohnStoychev, GeorgiHardy, John GeorgeStrauss, MathiasHarmandaris, VagelisStrehmel, BerndHarrisson, SimonStręk, TomaszHashidzume, AkihitoStringaro, AnnaritaHashimoto, MasanoriStrzemiecka, BeataHassager, OleStumpe, JoachimHassan, Mohammad K.Stylianakis, MinasHassanin, HanyStylianakis, Minas M.Hattori, YoshihideSu, An-ChungHatzikiriakos, Savvas G.Su, JunfengHaurie, LaiaSu, YanjieHayashi, FumitakaSu, ZhiqiangHayes, Douglas G.Su, ZhouchengHe, GuanjieSudha, P.N.He, HongliangSudo, AtsushiHe, JieSuemune, IkuoHe, QingsongSuen, Maw-CherngHe, ShaojianSugawa, KosukeHe, Wei-DongSugimoto, TomokoHe, WeilueSuh, Won HyukHe, WenhanSujecki, SlawomirHe, ZhongqiSukhikh, TaisiyaHeeley, Ellen LSumboja, AfriyantiHegde, MarutiSun, Bao AnHegmann, EldaSun, DiHeinrich, GertSun, HaoHeise, Herbert MichaelSun, JiazhenHejna, AleksanderSun, JingjingHellweg, ThomasSun, JingyaoHelmi, ArashSun, LemingHelseth, LarsSun, ShaolongHemraz, Usha D.Sun, XiuxuanHenczka, MarekSun, YingjieHenderson, Luke C.Sundaram, PaulHenriksen, Niel M.Suplicz, AndrasHermerschmidt, FelixSurace, RossellaHermida-Merino, DanielSuragani Venu, Lalith B.Hernandez-Montelongo, JacoboSuraneni, PrannoyHerrera-Ordonez, JorgeSurmeneva, MariaHerres-Pawlis, SonjaSutherland, TaraHesebeck, OlafSuzurikawa, JunHesp, Simon A.M.Svetlana, Tretsiakova-McNallyHevey, RachelSvoboda, PetrHevus, IvanSwamy, GaneshHeydari Gharahcheshmeh, MeysamSylvestre, AlainHeymann, MichaelSzczurek, AndrzejHideto, TsujiSzekalska, MartaHiejima, YusukeSzekely, GyorgyHigaki, YujiSzeleszczuk, lukaszHildner, RichardSzewczyk, RomanHiorns., RogerSzilagyi, AndrasHippler, RainerSzlęk, JakubHirai, TomoyasuSzolnoki, BeataHisada, KenjiSzostak, MarekHizal, GürkanSzperlich, PiotrHiziroglu, SalimSzubert, KarolHo, Chia-cheSzukalski, AdamHo, Ko-ShanSzurgacz, DawidHo, Wen-JengSzymanska-Chargot, M.Hobbs, Christopher E.Szymanski, RyszardHolze, RudolfSzymborski, TomaszHomaeigohar, ShahinSzymczyk, AnnaHonda, YoshitomoTabata, MakotoHong, Kyung HwaTaboryski, Rafael J.Hong, LiangTaddei, PaolaHong, SukjoonTaflove, AllenHong, TaoTaghiyari, Hamid RezaHongbing, DengTaguet, AurelieHonziCek, JanTaheri, ShimaHooper, Justin B.Taieb, AouakHorbach, JürgenTakai, MadokaHorrocks, RichardTakaya, TomohisaHorsky, JiřiTakayoshi, SuzukiHorvai, GeorgeTakehara, MunenoriHorvath, JuditTakemoto, HiroyasuHoskins, ClareTakeno, HiroyukiHossain, MokarramTakeoka, ShinjiHosseini Nejad, ElhamTakeshita, SatoruHosseinpourpia, RezaTakeuchi, DaisukeHou, ZhaoshengTamai, ToshiyukiHoujou, HirohikoTamm, TarmoHowell, BobTamura, AtsushiHowlin, BrendanTan, Chin YawHsiao, Pai-YiTan, GuoxinHsiao, Sheng-HueiTan, HongHsu, Fu-YinTan, ZhijianHsu, Po-ChunTanaka, RyoHu, AnmingTanaka, TomonariHu, GuangTang, BaokunHu, JingjieTang, LongtengHu, YangTang, Ren-ChengHu, YujinTang, YouhongHuang, CaoxingTang, YunlongHuang, Chien-LinTao, FeifeiHuang, Chih-FengTappa, KarthikHuang, Chi-HsienTappan, Bryce C.Huang, Chun-JenTarabanko, Valery E.Huang, Haw-MingTarbuk, AnitaHuang, JianyingTaresco, VincenzoHuang, JingTarres, QuimHuang, JingyuTavares, Maria Inês BrunoHuang, LiangliangTayeb, Ali H.Huang, Ming-ShyanTaylor-Pashow, KathrynHuang, QijinTayo, LemmeulHuang, WeiTcherdyntsev, Victor V.Huang, WeiminTefferi, MattewosHuang, XiaoyuTeixeira, Marcos F.S.Huang, YifengTejedor, Jose Antonio GomezHuang, YonghuiTelegdi, JuditHuang, YuchengTellez, CarlosHuang, YugangTelukutla, Srinivasa ReddyHuang, ZhifengTenhu, HeikkiHuerta-Angeles, GloriaTenorio-Alfonso, AdrianHughes-Riley, TheodoreTeramoto, HidetoshiHuijgen, WouterTeramoto, NaozumiHumpolicek, PetrTeramoto, YoshikuniHung, Chin-ChuanTerao, KenHung, Yung-TseTerasawa, NaohiroHuntosova, VeronikaTercjak, AgnieszkaHuo, QinghuanTerekhova, Irina V.Hussain, GhulamTervoort, TheoHutchinson, RobinTerzopoulou, ZoeHwang, SungyeonTerzopoulou, ZoiHwang, Ye-JinThakur, Vijay KumarHwang, YongsungTheodorou, DorosHyun, KyuTherias, SandrineIanchis, RalucaThitsartarn, WarintornIandolo, DonataThomas, SabuIatrou, HermisThompson, Richard L.Iconaru, Simona LilianaThuault, AnthonyIda, ShoheiTian, ZhitingIfelebuegu, AugustineTiera, MarcioIftekhar, SidraTing, Jeffrey M.Ikegami, TakahisaTobita, HidetakaIkehara, KenjiTofan, LaviniaIlg, PatrickTohmura, Shin-ichiroIllangakoon, U. ErankaToikka, AlexanderIllarramendi, Maria AssuncionTokarska, MagdalenaIllés, BalázsToldy, AndreaIlles, ErzsebetTomaszewska, EwaIllescas, JavierTomlinson, AimeeImamura, HiroshiTomšic, BrigitaInagaki, NorihiroTondi, GianlucaInomata, KatsuhiroTonelli, Alan EdwardIoelovich, MichaelTopuz, FuatIonita, PetreTorino, EnzaIordache, FlorinTornabene, FrancescoIordanskii, Alexey L.Török, TamasIqbal, Hafiz M. N.Torrado, Juan J.Irie, MasaoTorres Marques, AntonioIrusta, LourdesTorres Vaamonde, Jose EnriqueIsaeva, Vera I.Torres-Giner, SergioIsasi, Jose RamonTorres-Lagares, DanielIshige, RyoheiTorres-Ramirez, EduardoIshikawa, RyoTosello, GuidoIskierko, ZofiaToshio, KoizumiIslamoglu, TimurTotaro, GraziaIsmail, AhmedToth, AgotaIsmail, FlissToubal, LotfiIsoda, KyosukeTrabesinger, SigitaIsola, GaetanoTrache, DjalalIsono, TakuyaTrakakis, GeorgeIsreb, AbdullahTrcek, JanjaIssi, Jean-PaulTriboni, Eduardo R.Ito, SeigoTrifol, JonItoh, TakahitoTrimmel, GregorIvan, BelaTrindade, AnaIwan, AgnieszkaTritto, IncoronataIwko, JacekTrivedi, VivekIzawa, HironoriTrosien, SimonJacak, JaroslawTrotta, FrancescoJacquel, NicolasTrotta, GianlucaJahandar Lashaki, MasoudTrovato, FabioJahangir Alam, S.Trukhanov, AlexeiJaiswal, AmitTruong, Nghia P.Jajam, KailashTruskey, George A.Jakobik - Kolon, AgataTrusso Sfrazzetto, GiuseppeJamalabadi, Mohammad Yaghoub AbdollahzadehTsai, Chen-YenJames, RobinsonTsai, Chia-Hung DylanJamroz, PiotrTsai, Shiao-WenJanas, DawidTsai, Wei-BorJanczarek, MonikaTsakalof, AndreasJankauskaite, VirginijaTsalikis, DimitriosJanovak, LaszloTsarkova, Larisa A.Jansen, JohannesTseng, Hui-hsinJansen, K.M.B.Tsoi, James Kit-HonJanuszewski, RafalTsuchiya, KousukeJara, PaulTsuji, HidetoJarosz, TomaszTsukada, SatoruJastrzab, RenataTsurumaki, AkikoJastrzębska, MalgorzataTsutsui, MakusuJaszcz, KatarzynaTu, GuoliJauregui, Juan CarlosTuma, DirkJavaherdashti, RezaTunesi, MartaJavier, Sanchez-NievesTung, Shih-HuangJayakumar, RangasamyTurco, AntonioJayamani, ElammaranTuzimski, TomaszJedynak, KatarzynaTyliszczak, BozenaJeeyoung, YooTylkowski, BartoszJelliss, PaulTzoumanekas, ChristosJenkins, M. J.Tzounis, LazarosJeon, HyeonyeolUdalov, OlegJeon, Jong-RokUddin, MohammadJeon, Ju-WonUehara, TohruJeon, MinhyonUetani, KojiroJeong, Hoon EuiUgur, ŞaziyeJerman, IvanUma, KasimayanJesionowski, TeofilUmer, RehanJesson, DavidUnno, MasafumiJha, PrateekUppulury, KarthikJi, JunhuiUrakawa, OsamuJi, TuoUshakov, Nikolay M.Jia, BaoVadahanambi, SridharJia, ZhongfanVahabi, HenriJian, RongkunVaidyanathan, SriramJian, XigaoVaikuntanathan, VisakhJiang, ChengVaitkus, SauliusJiang, JiahuanValadares, LeonardoJiang, JunkeValerga Puerta, Ana PilarJiang, LaiValles-Lluch, A.Jiang, SaihuaVan Der Voort, PascalJiang, ShaohuaVan Erp, PietJiang, YanbingVan Steenberge, Paul Hm M.Jiang, ZaixingVanic, ZeljkaJiao, XuechenVankayala, RavirajJicsinszky, LaszloVaradi, CsabaJin, Hyeong MinVaradi, GyörgyiJing, QingshenVaradwaj, Pradeep R.Johanson, UrmasVashist, ArtiJohlitz, MichealVasile, CorneliaJoly-Duhamel, ChristineVasile, SimonaJones, ChrisVasiljević, JelenaJones, CliffVattikuti, Surya Veerendra PrabhakarJopek, HubertVazquez Sanchez, DanielJordan, AlexVecchione, Dr RaffaeleJoseph, J. MarcinkoVecino, XanelJoshi, NiravkumarVeiga, Maria-DoloresJulian, FernandoVelasco, Jose IgnacioJung, HyunVelea, Marian NicolaeJung, JaehanVenditti, IoleJung, Kyung-HyeVenkataraman, ShrinivasJung, SeunhoVenkatesan, JayachandranJung, Young HoonVenugopal, DhamodharanJunkers, TanjaVera Graziano, RicardoJuodkazis, SauliusVerduzco, RafaelJurga, StefanVernardou, DimitraJůza, JosefVesel, AlenkaKabir, AbuzarVetvicka, VaclavKaczmarek, HalinaVicaria, Jose M.Kadimisetty, KarteekVieregg, JeffreyKadokawa, Jun-ichiVihar, BoštjanKafarski, PawelVijayaraghavan, VenkateshKahol, PawanVikraman, DhanasekaranKairytė, AgnėVilà, AnnaKaja, SimonVilcakova, JarmilaKalantar-zadeh, KouroshVina Olay, JaimeKali, GergelyVinothkannan, MohanrajKaliva, MariaVirginie, PONSINETKalkan, A. KaanVitale, AlessandraKallitsis, Joannis K.Vithani, KapilkumarKalmar, JozsefVlachopoulos, JohnKalogiannis, Konstantinos G.Vodnar, Dan C.Kamal, Muhammad ShahzadVogiatzis, GeorgiosKamanina, Natalia V.Vohra, VarunKambhampati, Siva PramodhVoicu, Stefan IoanKamdem, DonatienVoigtmann, ThomasKaminaka, HironoriVoliani, ValerioKaminski, DanielVolker, KörstgensKaminski, RafalVoronov, AndriyKamitaka, YujiVorotyntsev, Ilya V.Kamperman, MarleenVoue, MichelKan, Chi-WaiVozzi, GiovanniKanazawa, ArihiroVrana, Nihal EnginKaneko, TakashiVrsaljko, DomagojKaneko, TatsuoVuluga, ZinaKanematsu, HideyukiWadgaonkar, PrakashKang, HyoWagland, StuartKang, Myung JooWajda, AleksandraKang, XinWakioka, MasayukiKangas, MichaelWalach, WojciechKao, Chai-LinWalęsa-Chorab, MonikaKaplan, AndreyWaller, Erik HagenKar, KamalWang, BinghaoKarahan, H. EnisWang, BoKarakas, SertacWang, ChenKarapanagiotis, IoannisWang, Chih-ChiehKaratrantos, ArgyriosWang, DanlingKarayiannis, NikosWang, ErgangKareiva, AivarasWang, FeiKarimzadeharani, AtefehWang, FuzhouKariz, MirkoWang, GangKar­jalainen, ErnoWang, HaoyuKarnati, Konda ReddyWang, HualinKarpinski, Tomasz M.Wang, JeffreyKaruppasamy, KWang, Jian-GanKaseem, MosabWang, Jian-WenKasmi, NejibWang, JikuiKatahira, RuiWang, JunKataoka, YusukeWang, LiangKatashima, TakuyaWang, LiliKauffman, Louis H.Wang, LingKaunas, RolandWang, LizhuKaur, AmandeepWang, LongKaushik, NagendraWang, Michael CaiKavita, KumariWang, QingguoKawakita, HidetakaWang, QiuguKazmer, David O.Wang, QunKazuhiro, ShikinakaWang, ShaokaiKe, XieWang, Shi-BinKee, ScholtenWang, ShifengKeki, SandorWang, SimonKelarakis, AntoniosWang, TaoKepinska, MartaWang, XiaoKerch, GarryWang, Xiao YongKhairulina, KaterynaWang, XiaodongKhan, AnzarWang, XiaofengKhan, Foysal ZahidWang, Xiaoguang (William)Khan, Omar F.Wang, XiaosongKhang, GilsonWang, XijunKharlampieva, EugeniaWang, XiluanKhattab, TawfikWang, XinKhmelev, Vladimir N.Wang, XuKhodadadi, Jay M.Wang, XuechuanKhorshidi, BehnamWang, YanbinKhoshnood, AbbasWang, Yen-ZenKhulbe, Kailash ChandraWang, YingKhurshid, ZohaibWang, YixiangKhwaldia, khaoulaWang, YongquanKiefer, RudolfWang, YongxiangKijenska, EwaWang, YuKikugawa, GotaWang, Yun-CheKim, Ae RhanWang, ZhaoKim, B. K.Wang, ZhaoxuKim, Byoung SuhkWang, ZhejunKim, Byung-JooWang, ZhiKim, ChoongikWang, ZhiningKim, Gue-HyunWang, ZhiweiKim, Hwan D.Wang, ZhongpengKim, Hyo JungWang, ZhuqingKim, Hyug-HanWani, Owies M.Kim, HyungwooWaskiewicz, SylwiaKim, Hyun-JoongWatanabe, WataruKim, HyunsungWatanabe, YoichiKim, IjungWebb, R. ChadKim, JaehyunWebber, GrantKim, JaewooWebste, Dean C. rKim, JaheonWeems, Andrew C.Kim, JooyounWei, BingKim, Kyung HoWei, GangKim, Kyung-MinWei, HuaKim, Mi-JeongWei, JiaKim, Min-JungWei, JunchaoKim, MyungwoongWei, KongchangKim, Nam KyeunWei, LiangmingKim, Pyung-HwanWei, QiangKim, Sang-HernWei, ZhongbaoKim, See JoWeis, MartinKim, SeokminWeiss, Clemens K.Kim, SokWen, ChunyiKim, Tae-HwanWen, YimeiKim, Tae-HyunWendel, RainerKim, WonhoWeng, ZhihuanKim, YunsangWhitmer, JonathanKing, LaurieWibowo, DavidKirillov, EvgueniWicklein, BerndKirillova, AlinaWie, Jeong JaeKirilov, PlamenWieckiewicz, MieszkoKiryukhin, Maxim V.Wieczorek, PiotrKiskan, BarisWieczorek, Wladyslaw G.Kiss, ImreWieleba, WojciechKitazawa, TakafumiWiessner, SvenKityk, IwanWilczyński, KrzysztofKlar, AgnesWillems, WimKlein, Lisa CWillerth, StephanieKlein, RolandWilsens, KarelKletschkowski, ThomasWinkler, RolandKlimov, Dmitry I.Winnacker, MalteKlotz, AlexWinnicka, KatarzynaKluczka, JoannaWischke, ChristianKnibbe, RuthWloch, MarcinKo, HyunhyupWohl, Christopher J.Ko, Jang MyounWojkiewicz, Jean-LucKodal, MehmetWolf, BettinaKoinkar, PankajWolff, MaxKoirala, NiranjanWöll, DominikKojdecki, Marek AndrzejWolny, StefanKokado, KentaWondraczek, KatrinKoller, MartinWong, Hsi-WuKomarov, Pavel V.Wong, Michael YinKomives, TamasWoo, EamorKondo, MioWoodward, Robert T.Kong, LingWorley, S.D.Kong, Ling BingWortmann, MartinKong, XiangruiWouters, PeterKonkolewicz, DominikWu, Chin-SanKonovalov, SergeyWu, Ding-taoKontoni, Denise-Penelope N.Wu, HongwuKoo, Hee-beomWu, Hung-ChinKoohbor, Behrad BehradWu, Jing-YunKopec, MaciejWu, Jyh-HorngKordatos, KonstantinosWu, KangningKorin, Eli J.Wu, Ming-ChungKorkees, FerasWu, PengKormanikova, EvaWu, TaoKorzeniewska, EwaWu, Tzi YiKorzhikov-Vlakh, ViktorWu, WangqingKoseva, NeliWu, XiangfaKostakis, GeorgeWu, YiboKostić, Aleksandar z.Wu, YunlongKostjuk, SergeiWu, YuqingKostka, LiborWu, Yu-TseKöstler, StefanWu, YuzhouKotelnikova, NinaWu, ZhangxiongKotsuchibashi, YoheiWu, ZongquanKou, JunlongWycisk, RyszardKoumoulos, Elias P.Wyman, IanKoutný, MarekWysokowski, MarcinKovaCević, DavorXia, ChangleiKovalcik, AdrianaXia, WenjieKowalczuk, MarekXiang, YuanKoyama, YasuhitoXiao, BoqiKozicki, MarekXiao, RuiKozielski, LucjanXiao, WeiKoziol, MateuszXie, HongfengKozlowska, JustynaXie, JingKragović, MilanXie, YunchuanKrasia-Christoforou, TheodoraXing, GuichuanKressler, JörgXiong, BijinKrishnan, SitaramanXiong, RengenKristak, LubosXu, AirongKröger, MartinXu, BaipingKrol-Kilińska, zanetaXu, BingangKruger, Jacob S.Xu, ChunfuKrupa-Kozak, UrszulaXu, DongyanKrystofiak, TomaszXu, FengKuang, TairongXu, FuchaoKubiak, TomaszXu, JiaxiKubo, MasatakaXu, Jia-ZhuangKucinski, KrzysztofXu, JunKuckling, DirkXu, LanKudaibergenov, SarkytXu, LuKuddannaya, ShreyasXu, TaoKudo, KazuakiXu, Xiao-DingKugler, SzymonXu, XiaoxueKujawa, JoannaXu, XiuruKujawski, WojciechXu, YitingKukula-Koch, WirginiaXu, ZhiweiKulichikhin, ValeryXuan, ShouhuKumanek, BogumilaXue, JiajiaKumar, AnujXue, QingzhongKumar, AnupamaYadav, HemrajKumar, AvneeshYadav, MithileshKumar, BrajeshYadavalli, Nataraja SekharKumar, PankajYalcinkaya, FatmaKumar, PradeepYamada, KazushiKumar, VipinYamaguchi, TetsuoKumeria, TusharYamamoto, EisukeKuncova, GabrielaYamato, TakehikoKunetsov, VladimirYamauchi, YusukeKunioka, MasaoYan, DayunKunitake, MasashiYan, JinlongKunli, GohYan, LiboKuo, Pei-YuYan, Li-BoKuo, ShiaoweiYan, NingKuo, Tsung-RongYan, ShoukeKupka, TeobaldYan, Zhi-ChaoKurańska, M.Yanase, TakashiKurańska, MariaYang, ChengKurc, BeataYang, Chen-IKurcok, PiotrYang, ChuanfangKuřitka, IvoYang, GuihuaKuzuya, AkinoriYang, HongyuKwiecień, IwonaYang, HsiharngKwon, Jae SungYang, HuKwon, Oh SeokYang, JianminKwon, Tae-YubYang, JingKwon, Young-WanYang, KejiaKyaw, Aung Ko KoYang, QuanlingKyle, StuartYang, RuihuaKyzas, GeorgeYang, ShuguangLa, Duc DuongYang, Ta-ILach, RalfYang, Teng-ChunLade, Harshad S.Yang, Tian-ZhiLadmiral, VincentYang, WeiLai, Jui-YangYang, YannanLai, YuekunYang, Yueh-Hsun KevinLaiarinandrasana, LucienYao, YangLamberti, LucianoYao, ZhitongLamberti, MarinaYao, Zi-JianLamnawar, KhalidYarlagadda, VenkataLamprou, DimitriosYasin, SohailLang, LihuiYazdi, ImanLange, HeikoYe, LongLanger, Jerzy JYelle, Daniel J.Lanterna, AnabelYeong, Wai YeeLanzi, MassimilianoYi, WeimingLappan, UweYildirim, AdemLaquintana, ValentinoYin, HengLarraneta, EnekoYing, HanzeLaschewsy, AndreYo, TanakaLaskowski, lukaszYobanny Reyes Lopez, SimonLasseuguette, ElsaYoo, Dong JinLattimer, BrianYoon, HyeonseokLau, Woei JyeYoshida, EriLaurent, ChristianYoshida, KentaroLaurenti, EnzoYoshihara, HiroshiLaurenzi, SusannaYoshihara, KumikoLaus, MicheleYoshikawa, KenichiLavandera, Jose LuisYoshikuni, TeramotoLavoine, NathalieYoshitake, HideakiLazzara, GiuseppeYou, JungmokLe, XiaoxiaYounes, HusamLe, ZaiyuanYoussry, MohamedLear, Benjamin J.Yu, BinLeBlanc, Roger MYu, Deng-GuangLebon, FredericYu, HaifengLecamp, LaurenceYu, Hai-YinLeckband, DeborahYu, Hsin HerLecomte, PhilippeYu, HuayangLedeţi, IonuţYu, JianLedwon, PrzemyslawYu, JiayiLee, Bae HoonYu, JingangLee, ChangwooYu, JinhongLee, Chia-HungYu, LeixiaoLee, Chih-HaoYu, LuLee, Jea UkYu, ShuiliLee, Jin WooYu, Tzyy LeonLee, Jin-KyunYu, WeilingLee, JongbokYu, Weiling (Connie)Lee, Ju-HyuckYu, XinjunLee, Jun SeokYu, YingfengLee, KangwonYu, YuxiLee, Kyung JinYu, ZhangLee, Li-TingYuan, ShangqinLee, Min WookYuan, ShengLee, PatrickYuan, ShushanLee, Pyung SooYuan, TianyuLee, Rong-HoYuan, YiLee, Seung GooYuguchi, YoshiakiLee, SeungaeYusa, Shin-ichiLee, Soo-HongZabihi, OmidLee, SunghoZafar, MuhammadLee, SunheeZafar, Muhammad SohailLee, Wen-YaZaharia, CatalinLee, WonohZakinyan, ArthurLee, Young-ChulZamboulis, AlexandraLee, Yung-chunZambrano, Maria De La LuzLee, Yun-Hanzamojć, KrzysztofLefèvre, ThierryZarabadi-Poor, PezhmanLeffler, HakonZaragoza Contreras, Erasto ArmandoLegrand, VincentZareba, Jan K.Legutko, StanislawZarejousheghani, MashaalahLehocky, MarianZarrelli, MauroLei, CaihongZarzar, Lauren D.Lelli, MorenoZarzyka, IwonaLemanowicz, MarcinZawierucha, IwonaLemli, BeataZdanowicz, MagdalenaLemos, FranciscoZechel, StefanLeneweit, GeroZednik, JiřiLeng, JingsongZelikman, EvgeniLeng, Yong XiangZelko, RomanaLeon, Gerardo A.Zeng, MinxiangLeon, LorraineZeng, QinghuaLeon, OriettaZeng, ShuwenLeonardi, FrancescaZeng, XiaoliangLeonat, LuciaZeng, YongchunLeon-Rivera, IsmaelZetterlund, PerLeporatti, StefanoZhai, LeiLesar, BoštjanZhai, TianruiLesov, IvanZhai, WentaoLetcher, ToddZhang, ChaoLeung, IvanhoeZhang, ChengLevacher, DanielZhang, ChunfuLevin, Oleg VladislavovichZhang, ChunjingLevine, Mindy S.Zhang, DaopengLewandowska, KatarzynaZhang, DeqingLi, AnlongZhang, FajunLI, BangjingZhang, FuLi, BinZhang, HaiLi, ChuanZhang, HailiangLi, DegangZhang, HanLi, GangZhang, HongboLi, HeZhang, HuiliangLi, Jiao JiaoZhang, JianyongLi, JiashenZhang, JieLi, JingZhang, LanyingLi, JinghaoZhang, LiqunLi, JuanZhang, MengLi, JunhuiZhang, MingzheLi, JunrongZhang, QiangqiangLi, JunshengZhang, QianqianLi, KaiZhang, QichunLi, LanZhang, RuoyuLi, LiangbinZhang, Shui-dongLi, LihuaZhang, WeiLi, LongyuZhang, WenxuLi, Lu HuaZhang, WujieLi, MeichunZhang, XiaoxingLi, MengZhang, XiaoyanLi, NanwenZhang, XiaoyongLi, PingZhang, Xuan-junLi, QiangZhang, XuefengLi, QingZhang, YanhuaLi, QuanxiangZhang, YanjieLi, RuosongZhang, YaohongLi, ShaojunZhang, YiyiLi, ShujunZhang, YongLi, Ting-TingZhang, YuLi, WeiZhang, YueLi, WenZhang, YumiaoLi, WenhaoZhang, ZeyuLi, WenyiZhang, ZhienLi, XinmingZhao, DongliangLi, XueZhao, FuliLi, Yeou-FongZhao, HanyingLi, YifanZhao, HongLi, YingZhao, JiLi, YiwenZhao, JianqingLi, YongjinZhao, KaiLi, YuanzuoZhao, LianfengLi, YuzhanZhao, LingLi, ZhaohuiZhao, ShouLi, ZhiZhao, Shu-NaLi, ZhiliangZhao, WeifengLi, ZhouZhao, XianhuiLiang, XingguoZhao, XinluoLiao, KinZhao, XuhuiLiao, Shu-ChuanZhao, YadongLiao, Ying-ChihZhao, YanLiaw, Chya-YanZhao, YapuLichtenhan, JosephZhao, Yong-BiaoLi-Destri, GiovanniZhao, YueLienkamp, KarenZhao, YunfengLikhanova, Natalya V.Zhao, ZhanLikozar, BlazZhao, ZhenghangLim, Chung-SunZhao, ZongminLim, Dong-JinZheng, BocongLim, Eun-KyungZheng, DafengLim, JiseokZheng, QingbinLim, SoomanZheng, SixuLima, Sofia CostaZheng, XintingLin, BorenZheng, YangLin, Ching HsuanZhong, LinxinLin, Chun-RongZhong, TuhuaLin, Hung-YinZhou, AoLin, JiaZhou, ChangluLin, Jia-HorngZhou, DezhongLin, Jun-HongZhou, DingyingLin, Kuo-ShyanZhou, DongfangLin, XiaojieZhou, JianhuaLin, XiaoshanZhou, KunLin, Yang-weiZhou, LiLin, ZhiweiZhou, ShengtaiLinares, JoseZhou, TaoLing, JunZhou, Wen-JingLinko, VeikkoZhou, XingpingLintinen, KalleZhou, Ying-guoLinul, EmanoilZhou, YiqunLionetto, FrancescaZhou, ZhengpingLiotier, Pierre-JacquesZhou, ZhuxianLipscomb, GlennZhu, CaihongLiras, MartaZhu, ChongyuLis Arias, Manuel J.Zhu, HuieLisak, GrzegorzZhu, JieLiso, VincenzoZhu, JuanjuanLiszkowska, JoannaZhu, TianyuLiu, BaicangZhu, ZhigangLiu, BaiquanZhukov, IgorLiu, BaojiangZiąbka, MagdalenaLiu, BoZiats, Nicholas P.Liu, Bo-TauZidek, JanLiu, Chao-LinZiegler-Borowska, MartaLiu, DanZille, AndreaLiu, FeiZinchenko, AnatolyLiu, Hongshunzmudzki, JaroslawLiu, HongyuZoltan-Istvan, SzaboLiu, JianZsuga, MiklosLiu, JiuxuZubitur, Manoli M.Liu, Jui-HsiangZukowski, WitoldLiu, JunZumstein, Michael ThomasLiu, LibinZuo, JianLiu, MinghuaZuorro, AntonioLiu, PengfeiZuppella, PaolaLiu, PengqingZuppolini, SimonaLiu, Ru


